# Impacts and Implications for Advancing in Environmental Knowledge in Hospitality Industry in COVID Society: a Bibliometric Analysis

**DOI:** 10.1007/s13132-022-00910-5

**Published:** 2022-01-26

**Authors:** Aurora Martínez-Martínez, Juan-Gabriel Cegarra-Navarro, Manuel-Jesús Cobo, Tiphaine de Valon

**Affiliations:** 1 Universidad Politécnica de Cartagena, Cartagena, Spain; 2 Universidad Cádiz, Cádiz, Spain; 3 Coventry University, Coventry, UK

**Keywords:** Bibliometric, Environmental knowledge, Hospitality, Knowledge orchestration, Tourism

## Abstract

Environmental knowledge is attracting interest in the area of sustainability due to the importance of both the environment and knowledge. As tourism is one of the biggest employers and sectors of economic development, environmental knowledge in hospitality represents a worldwide challenge. The present study aims to provide a clear understanding of the impacts and implications of environmental knowledge in the hospitality industry in a COVID society, taking into account its general areas of evolution through a systematic review methodology using a bibliographic database over time (26 years). We reviewed 944 documents collected from the Web of Science (WoS) Core Collection database and analysed them using the Science Mapping Analysis Software Tool (SciMAT). In a world in which the environment is more deteriorated, it is important to be aware of the advance in environmental knowledge to take care of it and eliminate environmental degradation. This study adds value to the orchestration of knowledge by focusing on predictors that impact environmental knowledge. The results identify the development status and leading trends in environmental knowledge research to fall in love with the future in a COVID society. Falling in love with the future is possible in the hospitality industry.

## Introduction

It is known that we have lost a lot due to COVID-19, the most important people that we will not see again. We have the option to cry or do everything we can to take care of the world and those we love. Why not focus on the opportunities posed by the COVID society in the hospitality industry? The mushrooming of COVID-19 related to the hospitality industry provides many opportunities to study and develop business. It is an excellent moment for environmental learning in the hospitality industry because stakeholders expect new skills, knowledge, capabilities and qualifications (Constantin Bratianu & Bejinaru, 2021; Sigala, 2020).

Over the past three decades, scholars have increasingly come to view knowledge as one of the most important resources for successful organisations in the contemporary socioeconomic landscape (Akhavan et al., 2016). Knowledge management (KM) has evolved and expanded in recent years (Bolisani & Bratianu, 2018; Cegarra-Navarro et al., 2015; Martínez-Martínez et al., 2018) in organisations.

Companies have had to address different issues dealing with economic, social, environmental and health concerns. It is well known that natural resources are deteriorating, and action must be taken now. The United Nations (UN) 2030 agenda for sustainable development urgently calls on governments, companies and individuals to make a transition to sustainable business models and lifestyles (United Nations, 2020). In this regard, the application of environmental knowledge has attracted the attention of different stakeholders, organisations and society as a whole (Cegarra-Navarro et al., 2010; Dumay et al., 2016; Martínez-Martínez et al., 2021).

Environmental knowledge in hospitality is a fundamental area that adds value to organisations by helping them achieve their goals. This helps to strengthen the capacity of organisations to create competitive advantages and care for the environment (Martinez-Martinez et al., 2019; Martínez-Martínez et al., 2015; Nieves & Haller, 2014).

Few areas have attracted the proposal of as many theories as environmental knowledge and its management. Despite this diversity, this study identifies several issues, which continue to challenge theoretical explorations of environmental knowledge in the hospitality industry. This study proposes a framework that describes the publications on environmental knowledge in the hospitality industry during the last 26 years. Therefore, emphasis is placed on possible ways to orchestrate flows of environmental knowledge in such a way as to maximise its potential value for organisations, while maximising the implicit and explicit benefits for other stakeholders. In doing so, this study aims to contribute to the literature by answering these questions:

- RQ1. Are publications on environmental knowledge in the hospitality industry growing?
- RQ2. What is the productivity of journals publishing papers on environmental knowledge in the hospitality industry? Which journals comprise the core of the field? What are the characteristics of the most cited papers in the field?
- RQ3: What are the main application domains?
- Q4. What are the main research gaps and upcoming research trends?

This paper adds to existing research in three critical aspects. Firstly, it provides a first and clear understanding of ongoing research by examining the current status, development and future research directions in the field of environmental knowledge in hospitality over the period 1994–2020 as a 26-year longitudinal study. Secondly, this study provides the current status, development and future research directions in the field. Thirdly, this work contributes to presenting a more frequent denomination of the keywords in the academic literature; in other words, this study contributes to offering an orchestration of the knowledge in the field. Finally, this study contributes to academia, professionals and governments’ theoretical and practical implications.

This work is organised as follows. “Introduction” introduces the theoretical background, while “Methodology: Science Mapping Analysis” explains how the methodology was used to develop this research. “A Bibliometric Analysis of Environmental Knowledge in the Hospitality Industry” describes the performance of the bibliometric analysis. “Science Mapping Analysis, Findings and Evolution Map of Environmental Knowledge in the Hospitality Industry” develops the science mapping analysis and the conceptual evolution map, and “Discussion” develops the discussion. Finally, “Conclusion, Limitations and Future Perspectives for Research” provides the conclusion, limitations of this study and future lines of research.

## The Theoretical Background of Environmental Knowledge Management in the Hospitality Industry

Hospitality is an important industry worldwide. In 2019, the hospitality industry contributed US$8.9 trillion to the world’s GDP, 10.3% of the global GDP and 330 million jobs or, in other words, 1 in 10 jobs worldwide (WTTC, 2020). Nowadays, the hospitality industry is facing changes due to the coronavirus (COVID-19) pandemic that has swept across the globe and has not only had a significant impact on public health but also severely affected one of the linchpins of the global economy, hospitality (Dias et al., 2020; Jones & Comfort, 2020; Rivera, 2020). This is, however, an excellent opportunity to reconsider the renovation of the hospitality industry to be better aligned to the UN’s Sustainable Development Goals (SDGs) (Gössling et al., 2021). The existing measures to fight COVID-19 have increased the generation of plastic waste, among other pollutants (Filimonau, 2020; Patrício Silva et al., 2020). The potential effects on the environment might persist and worsen along with the health emergencies caused by the pandemic. For instance, although these are preliminary estimations, there is even fear that pollution may facilitate the transmission of coronaviruses (Espejo et al., 2020; Qu et al., 2020). It appears clear that the hospitality industry must face new challenges and consider new economic, social (health) and environmental aspects to achieve its goals and create competitive advantages (Jones & Comfort, 2020). Sustainability is a challenge for this industry that should be addressed in its activities (Filimonau, 2020).

Sustainability in hospitality has attracted the attention of researchers and practitioners from differing perspectives, such as environmental management systems (Chan, 2009; Font, 2002), firm performance (Martínez-Martínez et al., 2015), destination performance (Jiang & McCabe, 2021), air pollution (Mulcahy et al., 2005), customer loyalty (Ja-Shen et al., 2017), corporate social responsibility (de Grosbois, 2012; Zaragoza-Sáez et al., 2020) and employees (Bohdanowicz et al., 2011; Pham et al., 2020; Zientara & Zamojska, 2018). However, the application of sustainability to the area of knowledge management has not been previously explored.

Knowledge is commonly understood to condition a person’s behaviour (Frick et al., 2004), and knowledge management can be helpful in the proper detection, advance, use and distribution of important knowledge, which, in turn, can improve an organisation’s sustainability (Durst & Zieba, 2020). Knowledge management can be used to manage uncertainty, turbulence and dynamics (Alexandru et al., 2020), to plan strategies and to contribute to decision-making processes (Bratianu et al., 2020; Guerras-Martín et al., 2014). It can be seen as continuous learning and adaptation to meet the needs and opportunities that dynamically emerge from daily situations (Bolisani & Bratianu, 2017; Cegarra-Navarro et al., 2018). In addition, knowledge management can contribute to solving problems or mitigating barriers through continuous learning (Sánchez-Polo et al., 2019). Furthermore, knowledge management can create an open-minded context through the exploration and exploitation of knowledge (Wensley et al., 2011) and it can contribute to improving sustainability and developing environmental knowledge.

The concept of environmental knowledge is defined as the use of knowledge management strategies, tools and techniques to create, share and reuse tacit and explicit knowledge resources related to the environment and its protection (Martinez-Martinez et al., 2019b). The management of environmental knowledge is a resource that can improve sustainability in the hospitality industry, and the environmental knowledge structures in this industry can promote changes in stakeholders’ behaviour regarding sustainability and the quality of life all over the world (Kornilaki et al., 2019; Martínez-Martínez et al., 2021). To answer the research questions of this study, we analyse the past and current status of environmental knowledge in the hospitality industry, and we explore its evolution. This is an interesting and unexplored field which should be covered.

The next section develops the methodology of this research and provides an overview of the bibliometric analysis.

## Methodology: Science Mapping Analysis

The main purpose of this analysis is to map the thematic perspectives of environmental knowledge in the hospitality field. The bibliometric analyses will help to clarify the past and current status of these studies and develop future lines of research though its indicators (Gaviria-Marin et al., 2018). The SciMAT software tool (Cobo et al., 2012) was used to detect the main research topics through a science mapping analysis (Cobo et al., 2011a, b) based on longitudinal co-word bibliographic networks.

SciMAT was chosen because it includes a robust methodology based on bibliometric indicators and bibliographic networks (Cobo et al., 2011a).

**Fig. 1:**
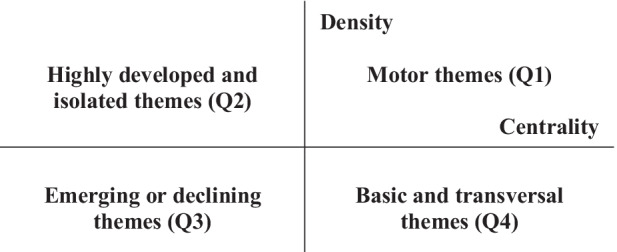
Description of the strategic diagram. Source: Cobo et al. (2012)

The conceptual science mapping analysis was conducted with SciMAT, using the four-stage approach proposed by Cobo et al. (2011a):

1. Findings of the investigation topics. Keywords from the research papers for each period build a network based on keyword co-occurrence. The nodes represent the different keywords, and there is an edge between two connections if both keywords appear in a set of papers. Then, in each period, a clustering algorithm is applied to a normalised co-word network to distinguish the study themes.
2. Visualising research themes and the thematic network. Figure 1 shows a graphic description of the strategic diagram according to two dimensions: the density and centrality of the themes. The most important and basic themes for structuring a research topic are in the upper-right quadrant (Q1). Themes in the upper-left quadrant are considered to be highly developed, but they are isolated themes (Q2). In the lower-right quadrant are the basic and transversal themes (Q4), and finally, declining or emerging themes are in the lower left (Q3). This quadrant is important for the identification of possible emerging themes of future research.
3. Discovery of thematic areas. The periods show the evolution of the themes in their research fields, their origins and their interrelationships.
4. Performance analysis. In this phase, the impact of each topic is measured using bibliometric indicators.

### Data Collection

This work analyses scientific documents in the Web of Science (WoS) Core Collection database. WoS is a comprehensive search engine that provides full results for accurate analysis since it contains a wide range of detailed information about every document. The search was carried out in September 2020, and the research criteria is (TS = ((“environmental knowledge” OR “green knowledge” OR “environment knowledge” OR “environmental” OR “green” OR “Sustainability knowledge” OR “sustainability”) AND (“hospitality”))). Our advanced query retrieved 1126 initial results. However, the corpus was further restricted to refine and delimit the search by using some exclusion criteria: publication year (1994–2020 period), document types (article) and language (English). And the options were refined by: DOCUMENT TYPES: (ARTICLE) and [excluding] DOCUMENT TYPES: (BOOK CHAPTER OR PROCEEDINGS PAPER). Timespan: 1994–2020. Indexes: SCI-EXPANDED, SSCI, A&HCI, CPCI-S, CPCI-SSH, BKCI-S, BKCI-SSH, ESCI, CCR-EXPANDED, IC. This process resulted in a total of 944 papers from the WoS Core Collection.

The whole corpus was divided into three consecutive time periods using the period manager of SciMAT to determine the evolution of sustainable knowledge in the hospitality industry. Ideally, the whole period should be divided into an equative number of years; however, in this case, the whole period was divided into comparable periods in terms of production, following indications by the following authors (Cobo et al., 2012; Garcia-Buendia et al., 2020). The complete period (1994–2020) was divided into three subperiods for 1994–2010, 2011–2015 and 2016–2020, with 119, 173 and 652 respective publications in each established period, respectively. This study analysed a period of 26 years. The first period is longer than the other two periods because this was necessary to enable the science mapping analysis to detect the main research themes. This method of dividing the entire period allowed us to observe the evolution of the topic *environmental knowledge in hospitality* from its beginnings to the most recent research. While the first period included the origins of the field, the second shows the evolution and the third period covers new trends in the topic. A particularity of the fields of knowledge and sustainability shows that the three periods demonstrated an interest in health. This division provided the evolution of the topic from its beginnings to the most recent research.

## A Bibliometric Analysis of Environmental Knowledge in the Hospitality Industry

This section describes the evolution of environmental knowledge in hospitality in terms of publication distribution, citations and impact through the analysis of the following bibliometric indicators: published papers, citations, journal impact factors, most productive countries and most cited papers and their authors. The bibliometric study is designed in two parts: (1) production and citations and (2) production, journals, countries and research areas. This section analyses the evolution of environmental knowledge in hospitality to answer RQ1: Are publications on environmental knowledge in the hospitality industry growing? And RQ2: What is the productivity of journals publishing papers on environmental knowledge in the hospitality industry? Which journals comprise the core of the field? What are the characteristics of the most cited papers in the field?

### Publications and Citations

**Fig. 2:**
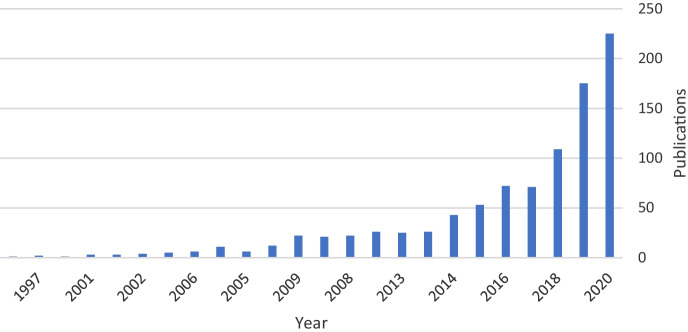
Distribution of publications by year (1994–2020). Source: own elaboration

Three stages can be observed in the development of the publications under study since the first publication in 1994 (Fig. 2). While period 1 (1994–2010) shows very slow growth, period 2 (2011–2015) shows increasing interest in the subject. Period 3 shows that the number of publications has increased and that the number of publications grew substantially during this period (2016–2020). Figure 1 shows that the field analysed in this bibliometric study is of current interest and that this interest is increasing. This continuing trend could imply that the number of publications could continue to grow in the future.

Table 1 shows the ten countries that were the most productive in environmental knowledge in hospitality during the period 1994 to 2020. This information is based on the filiation of the authors. Countries with publications are associated with a minimum of one author. The third period, 2016–2020, is the most productive period during which increasing research development in the field was observed. In the first positions are the USA, Spain and England. They represent a cumulative percentage of 58.06%.

**Table 1:** Most productive countries in the field by period

Position	Countries/regions	Publications	% of 944	1994–2010	2011–2015	2016–2020
1	USA	210	28.23	38	52	120
2	Spain	124	16.67	8	24	92
3	England	98	13.17	10	12	76
4	People’s Republic of China	93	12.50	2	19	72
5	Australia	80	10.75	8	17	55
6	Italy	53	7.12	0	13	40
7	Taiwan	50	6.72	7	1	42
8	Turkey	45	6.05	2	1	42
9	Canada	32	4.30	4	8	20
10	South Korea	31	4.17	2	2	27

Source: own elaboration

The most cited items are shown in Table 2. Table 2 also shows the 10 top publications, total citations, journals, authors, titles of research, years and positions according to the most cited work. The main paper topics are corporate social responsibility, environmental certification, environmental management practices, decision-making for sustainability, examples of international hotel chain success in control of air pollution and tobacco and workers’ exposure to air pollution.

**Table 2:** The most cited items

Position	Title	Authors	Source title	Publication year	Total citations
1	Corporate social responsibility reporting by the global hotel industry: Commitment, initiatives and performance	de Grosbois, Danuta	*International Journal of Hospitality Management*	2012	221
2	Environmental certification in tourism and hospitality: progress, process and prospects	Font, X	*Tourism Management*	2002	187
3	Greening the service profit chain: The impact of environmental management practices	Kassinis, GI; Soteriou, AC	*Production and Operations Management*	2003	184
4	Going green: Decisional factors in small hospitality operations	Tzschentke, Nadia A.; Kirk, David; Lynch, Paul A	*International Journal of Hospitality Management*	2008	164
5	Legislation for smoke-free workplaces and health of bar workers in Ireland: before and after study	Allwright, S; Paul, G; Greiner, B; Mullally, BJ; Pursell, L; Kelly, A; Bonner, B; D'Eath, M; McConnell, B; McLaughlin, JP; O'Donovan, D; O'Kane, E; Perry, IJ	*BMJ-British Medical Journal*	2005	161
6	International hotel chains and environmental protection: an analysis of Hilton's we care! programme (Europe, 2006–2008)	Bohdanowicz, Paulina; Zientara, Piotr; Novotna, Emilie	*Journal of Sustainable Tourism*	2011	153
7	Respirable particles and carcinogens in the air of Delaware hospitality venues before and after a smoking ban	Repace, J	*Journal of Occupational and Environmental Medicine*	2004	152
8	Second-hand smoke exposure and risk following the Irish smoking ban: an assessment of salivary cotinine concentrations in hotel workers and air nicotine levels in bars	Mulcahy, M; Evans, DS; Hammond, SK; Repace, JL; Byrne, M	*Tobacco Control*	2005	143
9	Drivers and barriers of peer-to-peer accommodation stay—an exploratory study with American and Finnish travellers	Tussyadiah, Iis P.; Pesonen, Juho	*Current Issues in Tourism*	2018	141
10	Changes in hospitality workers' exposure to second-hand smoke following the implementation of New York's smoke-free law	Farrelly, MC; Nonnemaker, JM; Chou, R; Hyland, A; Peterson, KK; Bauer, UE	*Tobacco Control*	2005	133

Source: own elaboration

Table 3 lists the journals with a record quantity of publications on environmental knowledge in the hospitality industry. The columns give information about the record count of papers published and the representative percentages of their sources.

**Table 3:** Source titles

Position	Source titles	Record count	% of **944**
1	*International Journal of Contemporary Hospitality Management*	57	79.50
2	*Sustainability*	50	69.74
3	*International Journal of Hospitality Management*	49	68.34
4	*Journal Of Sustainable Tourism*	30	41.84
5	*Cornell Hospitality Quarterly*	16	22.32
6	*Journal Of Cleaner Production*	16	22,32
7	*Tobacco Control*	16	22.32
8	*Tourism Management*	15	20.92
9	*Worldwide Hospitality and Tourism Themes*	13	18.13
10	*Journal of Hospitality and Tourism Management*	11	15.34

Source: own elaboration

Table 4 presents the most relevant research areas and Web of Science categories during the period of 1994 to 2020 with their number of publications.

**Table 4:** Most relevant research areas and WoS categories (1994–2020)

	Research areas	Publications	WoS categories	Publications
1	Social sciences and other topics	343	Hospitality leisure sport tourism	333
2	Business economics	210	Management	167
3	Environmental sciences and ecology	155	Environmental sciences	122
4	Science technology and other topics	110	Green sustainable science technology	105
5	Public environmental occupational health	95	Public environmental occupational health	95
6	Engineering	38	Environmental studies	81
7	Sociology	23	Business	60
8	Substance abuse	22	Engineering environmental	28
9	Education and educational research	20	Sociology	23
10	Toxicology	18	Substance abuse	22

Source: own elaboration

## Science Mapping Analysis, Findings and Evolution Map of Environmental Knowledge in the Hospitality Industry

In this section, the science mapping analysis, findings and the relationships between the keywords and terms used in environmental knowledge in the hospitality field are explained. The section is organised into two interrelated parts: a content analysis of the works, findings and the evolution map of the thematic areas covered. This format can be used to detect the themes in environmental knowledge in the hospitality corpus for each of the three periods. The second part shows both the development of these investigation themes and the relationships between them for the total period of analysis. This contributes to determine gaps and identify possible lines of future research.

To achieve a better understanding of the findings of this bibliometric analysis, the arguments are organised into two sections. The first section groups the main theoretical concepts and relationships that focus on environmental knowledge in the hospitality industry. The second section presents the applied results found in this bibliometric analysis and articulates the relations with environmental knowledge and organisational learning in the hospitality industry.

It analyses the evolution of environmental knowledge in hospitality to answer RQ3: What are the main application domains?

### Findings and Strategic Diagrams

**Fig. 3:**
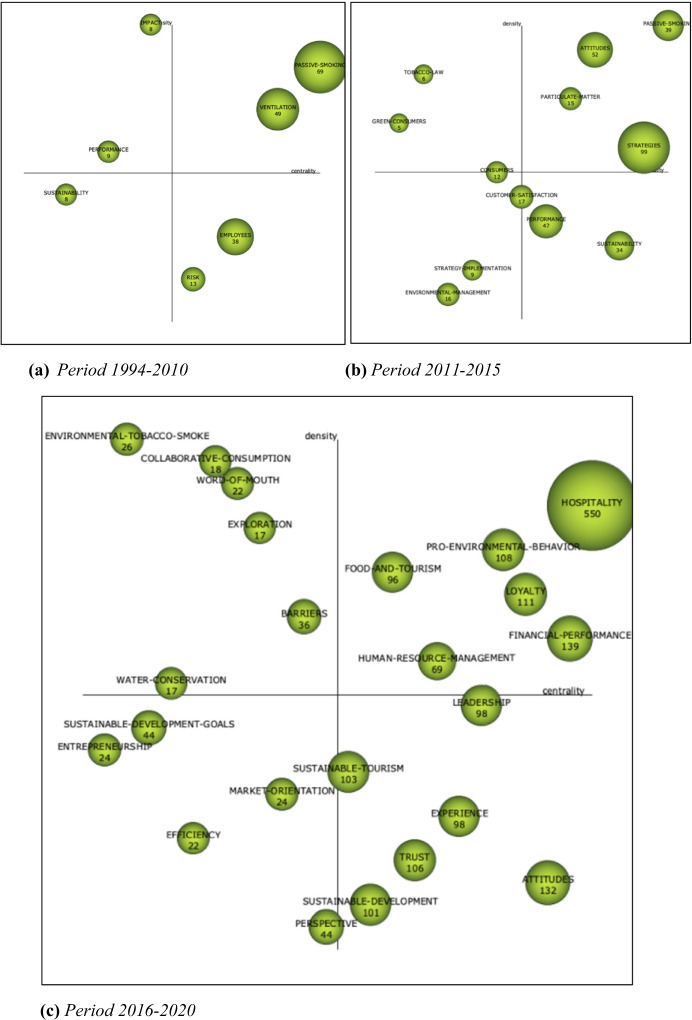
Strategic diagrams: **a** period 1994–2010; **b** period 2011–2015; **c** period 2016–2020

Figure 3 shows three strategic diagrams for the analysis of the most frequently treated themes in environmental knowledge in hospitality during the period of 1994 to 2020. The research themes in the strategic diagrams are associated with circles, and the size of the circles is related to the number of citations connected with them. In other words, it was modelled using the centrality and density measures. Centrality estimates the degree of network interaction by analysing the links between keywords inside and outside the network. Density considers internal thematic coherence by examining the links between keywords inside the network. In Fig. 1, the meaning of quadrants in the strategic diagram was explained. Strategic diagrams of the different periods a, b and c are explained below.

#### First Period (1994–2010)

As is shown Fig. 3a, the strategic diagram includes seven research themes related to environmental knowledge during this period, with three themes considered essential for their contribution to the evolution of the field. In other words, they are motor themes and basic and transversal themes: PASSIVE SMOKING and VENTILATION. The emerging or declining theme during this period 1994–2010 appears to be SUSTAINABILITY. Lastly, IMPACT and PERFORMANCE appear as highly developed, isolated themes.

Table 5 shows clusters of information from the period 1994 to 2010 related to the number of publications and h-indexes of the documents. Based on the number of citations and the h-index, the main research themes directly related with the research criteria of the field during the period 1994–2010 were as follows: PASSIVE SMOKING and VENTILATION.

**Table 5:** Performance of themes during 1994–2010

Theme (quadrant)	Documents	h-index
PASSIVE SMOKING (Q1)	69	28
VENTILATION (Q1)	49	26
EMPLOYEES (Q4)	38	20
RISK (4)	13	13
RISK (Q4)	12	12
IMPACT (2)	8	8
PERFORMANCE (2)	9	8
SUSTAINABILITY (3)	8	7

Source: own elaboration

PASSIVE SMOKING can be considered the most important research theme during the period 1994–2010 since it obtains the largest number of documents and the highest document h-index. It was an important health-related theme during the years when the problem of tobacco and its connection to cancer was increasing, and revelations concerning the terrible consequences for active and passive smokers were made (Chan, 2009). It is important to highlight that this motor theme appeared during the period 1994–2010, when awareness, prevention and information about the negative effects of tobacco on health began, and when more restrictive laws were implemented limiting the use of tobacco in public areas, which was a decision that impacted the hospitality industry.

VENTILATION is considered an important research theme during the period 1994–2010 since it has the second-largest number of documents indicated and the second-highest document h-index. Ventilation theme is connected with tobacco themes. However, both were highly researched themes and were studied by separated. The problem of tobacco and its effect on workplaces, passive smokers and laws were themes with a growing interest in seeking solutions with connection to the ventilation theme.

Finally, we indicate that the role of employees is important during this period for two reasons: one of them involves the negative effects of air pollution and is passive, and the other one is active, as employees are considered to be agents of knowledge and they can contribute to implementing healthier and more sustainable practices in hospitality (Font, 2002; Tzschentke et al., 2008).

#### Second Period (2011–2015)

According to the strategic diagram in Fig. 3b, nine study themes related to environmental knowledge in hospitality were identified during the second period. These are the five most important research themes, in other words, motor themes and basic and transversal themes. These motor themes during this period are PASSIVE SMOKING, LUNG CANCER, SUSTAINABILITY, PERFORMANCE, and HEALTH.

Another aspect of environmental knowledge in hospitality that appeared as a transversal or emerging theme was ATTITUDES. Lastly, RESOURCE-EFFICIENCY, FINE PARTICULATE MATTER and TOBACCO LAW appear as highly developed and isolated themes.

Table 6 shows clusters of information for the period 2010–2014 related to the number of publications and the h-index.

**Table 6:** Performance of themes during 2011–2015

Theme (quadrant)	Documents	h-index
STRATEGIES (Q1)	99	35
PERFORMANCE (Q4)	47	25
ATTITUDES (Q1)	52	25
SUSTAINABILITY(Q2)	34	17
PASSIVE-SMOKING (Q1)	39	12
CUSTOMER-SATISFACTION (Q4)/(Q3)	17	12
ENVIRONMENTAL-MANAGEMENT (Q3)	16	12
CONSUMERS (Q3)/ (Q2)	12	9
PARTICULATE-MATTER (Q1)	15	8
STRATEGY-IMPLEMENTATION (Q3)	9	6
GREEN-CONSUMERS (Q2)	5	5
TOBACCO-LAW (Q2)	6	3

Source: own elaboration

PASSIVE SMOKING, PARTICULATE-MATTER, ATTITUDES and STRATEGIES cover several aspects of the strategy to control the nexus of air pollution to hospitality workers. During this period (Q1), there was great interest in the transformation of the hospitality industry into a more environmentally aware sector. The strategies were aligned with environmental management systems, environmental knowledge and unlearning to be able to learn from past mistakes (Cegarra-Navarro et al., 2010).

During the period 2011–2015, SUSTAINABILITY is a basic and transversal theme, and some of the cluster networks in the studies are “competitiveness”, “ISO-14001”, “environmental certification”, “innovation” or “stakeholders”. There was a time when all kinds of activities were carried out without addressing sustainability issues, but this has fortunately changed. Progress has been made by a wide range of private, non-profit and public organisations in developing and measuring environmental knowledge. Their objective is to take care of the environment and promote sustainability in the hospitality industry through internationally coordinated processes of compliance, assessment and the achievement of common goals (Bohdanowicz et al., 2011; de Grosbois, 2012; Font, 2002; Sharma et al., 2021).

The basic and transversal theme PERFORMANCE is linked to market orientation, environmental responsibility, corporate social responsibility, environmental knowledge, environmental management, strategies and service quality. This theme includes some essential issues that should be considered when attempting to make the hospitality industry more sustainable (Cegarra-Navarro & Martínez-Martínez, 2010).

The last basic and transversal theme is CUSTOMER-SATISFACTION which is also considered as emerging theme. It is also considered an emerging issue along with CONSUMERS, STRATEGY-IMPLEMENTATION or ENVIRONMENTAL-MANAGEMENT.

In period 2, researchers were interested in studying TOBACCO LAW. Tobacco legislation is very restrictive, and this theme appears as a highly developed and isolated theme, and GREEN CONSUMERS was highly developed and isolated theme during the period 2011–2015.

#### Third Period (2016–2020)

As seen in the strategic diagram in Fig. 3c, twenty-one research themes related to environmental knowledge in hospitality were identified during the third period. There are twenty-three main research themes, in other words, motor themes and basic and transversal themes. The motor themes during this period are HOSPITALITY, FINANCIAL-PERFORMANCE, LOYALTY, PRO-ENVIRONMENTAL BEHAVIOUR, FOOD-AND-TOURISM and HUMAN-RESOURCE-MANAGEMENT.

The performance measures in Table 7 show that HOSPITALITY, FINANCIAL-PERFORMANCE, ATTITUDES, LOYALTY, PRO-ENVIRONMENTAL BEHAVIOUR, TRUST, SUSTAINABLE-TOURISM and SUSTAINABLE DEVELOPMENT are the themes with the largest numbers of papers.

**Table 7:** Performance of themes during 2016–2020

Theme (Quadrant)	Documents	h-index
HOSPITALITY (Q1)	550	33
PRO-ENVIRONMENTAL BEHAVIOUR (Q1)	108	21
FINANCIAL-PERFORMANCE (Q1)	139	21
LOYALTY (Q1)	111	21
ATTITUDES (Q4)	132	19
LEADERSHIP (Q4)	98	18
EXPERIENCE (Q4)	98	18
HUMAN-RESOURCE-MANAGEMENT (Q1)	69	17
TRUST (Q4)	106	17
SUSTAINABLE-DEVELOPMENT (Q4)	101	17
SUSTAINABLE-TOURISM (Q4)	103	17
FOOD-AND-TOURISM (Q1)	96	16
PERSPECTIVE (Q3)	44	11
EFFICIENCY (Q3)	22	11
BARRIERS (Q2)	36	9
MARKET-ORIENTATION (Q3)	24	9
ENTREPRENEURSHIP (Q3)	24	9
SUSTAINABLE-DEVELOPMENT-GOALS (Q3)	44	8
WORD-OF-MOUTH (Q2)	22	8
WATER-ORIENTATION (Q2)	17	8
EXPLORATION (Q2)	17	7
ENVIRONMENTAL-TOBACCO-SMOKE (Q2)	26	6
COLLABORATIVE-CONSUMPTION (Q2)	18	6

Source: own elaboration

For HOSPITALITY, the cluster networks are corporate social responsibility, management, performance, satisfaction, sustainability, tourism, behaviour, innovation, perceptions, strategies and environmental management. It is well known that the hospitality industry has represented an important percentage of the GDP worldwide in recent years. The hospitality industry is a major part of the world economy (Jones & Comfort, 2020) and provides livelihood to millions of workers and their families. Its activities are closely related to the environment (Omerzel, 2016).

For FINANCIAL-PERFORMANCE, the cluster networks are social norms, theory of planned behaviour, green hotel, intention, complexity, conservation, intrinsic motivation, planned behaviour, self-determination theory and pro-environmental behaviour (Omerzel, 2016).

The theme of LOYALTY is consolidated as a motor theme during this period. This research theme gains attention during this period, and its cluster networks are social responsibility, supply chain management, strategies, accountability, competitive advantage, dynamic capabilities, firm performance, financial performance and quality management. This theme has motivated different works from a common perspective of attitudes, competences and knowledge agents as drivers of environmental sustainability (Martinez-Martinez et al., 2019b).

For PRO-ENVIRONMENTAL-BEHAVIOR, the cluster networks are corporate-social-responsibility, management, performance, satisfaction, sustainability, innovation, perceptions and environmental-management (Martínez-Martínez et al., 2021; McTiernan et al., 2021; Warren et al., 2018).

The theme of FOOD AND TOURISM is motor theme during this period. This research theme gains attention during this period, and its cluster networks are green image, customer satisfaction, beliefs, attributes, motivations, emotion, service quality, perceived value and image. Eliminating food waste is one of the great challenges of our century.

For HUMAN RESOURCE MANAGEMENT, the cluster networks are job satisfaction, opportunities, stakeholder engagement, capabilities and leadership (Martínez-Martínez et al., 2019a).

The BARRIERS, ENVIRONMENTAL-TOBACCO-SMOKE, WORD-OF-MOUTH, COLLABORATIVE-CONSUMPTION, EXPLORATION and WATER-CONSERVATION themes were greatly developed during the period 2016–2020. Therefore, the thematic area “health” is an important theme in studies about environmental knowledge in hospitality (Mitova et al., 2016).

Finally, SUSTAINABLE-DEVELOPMENT-GOALS, ENTREPRENEURSHIP, MARKET-ORIENTATION and EFFICIENCY were emerging themes in this research criteria (Alegre & Berbegal-Mirabent, 2016). Policies related to sustainable development goals, consumers, entrepreneurship, job satisfaction and efficiency in the hospitality industry could be important themes in the future to cope with the changes brought on by COVID-19.

Table 7 shows information clusters for the period 2016–2020 related to the number of publications and the h-index.

### Conceptual Evolution Map

A second analysis was made focussing on the conceptual evolution of the core themes by considering the results of the content document analysis for the three periods. The themes of research, concepts and keywords that authors used evolved throughout the years. Where some new concepts appeared, others fell into disuse. Therefore, the keyword set used in each period provides information to determine whether the number of researched topics increased with emerging themes, whether it decreased with declining themes or whether the motor themes are the same. The Inclusion index was used in this study to track the evolution of the vocabulary in the field (Cobo et al., 2011a). This development found that research into environmental knowledge in hospitality was concentrated into four thematic areas: Health/Passive Smoking/Employees, Sustainable development, Pro-Environmental Behaviour and Customer Satisfaction. Table 8 shows the thematic areas.

**Table 8:** Performance of the thematic areas (1995–2019)

Thematic area	Documents	Citations	h-index
Sustainable development	714	9908	48
Pro-environmental behaviour	356	5012	39
Health/passive smoking/employees	160	4556	36
Customer satisfaction	290	4053	34

Source: own elaboration

The Health/Passive Smoking/Employees area is composed of important healthcare concepts that apply environmental knowledge to prevent cancer and mitigate or eliminate the negative effects of smoke in the workplace and risks in public hospitality spaces. Environmental knowledge is making important contributions to the field of health. For instance, researchers are interested in developing more sustainable and healthier workplaces (Drope & Chapman, 2001; Mitova et al., 2016). The area covers research about environmental knowledge applied to ventilation and fine-particulate matter. Findings in this area could be applied to different contexts, such as the pandemic crisis (Mulcahy et al., 2005). The work about Employees principally includes motor and transversal themes during the three periods. This area comprises research on environmental knowledge applied by workers in different models (implementation of certification, corporative social responsibility, knowledge sharing, skills, capabilities, etc.). The presence of links with sustainability in hospitality should be highlighted. The effect of environmental methods and knowledge agents mainly focusses on attitudes. It is also interesting to highlight that intellectual capital is crucial in this area (Martinez-Martinez et al., 2019a, b).

Sustainable development is composed of works such as exploration of environmental knowledge, orientation to market, leadership, entrepreneurship, engagement, social media, brand equality, commitment, consequences, customer loyalty, decision making or financial performance.

The area of Pro-Environmental Behaviour includes works focussing on pro-sustainability from the perspectives of stakeholders, businesses and society (Martínez-Martínez et al., 2021).

Customer Satisfaction is composed of works in which environmental knowledge strategies are a tool to distinguish companies from their competitors, thereby obtaining competitive advantages. The hospitality industry must change their procedures and routines to include more human, relational and structural capital to obtain customer satisfaction (Engström Truls et al., 2003). The research community is paying attention in this period to new topic as food waste or improvement of sustainable development goals.

**Fig. 4:**
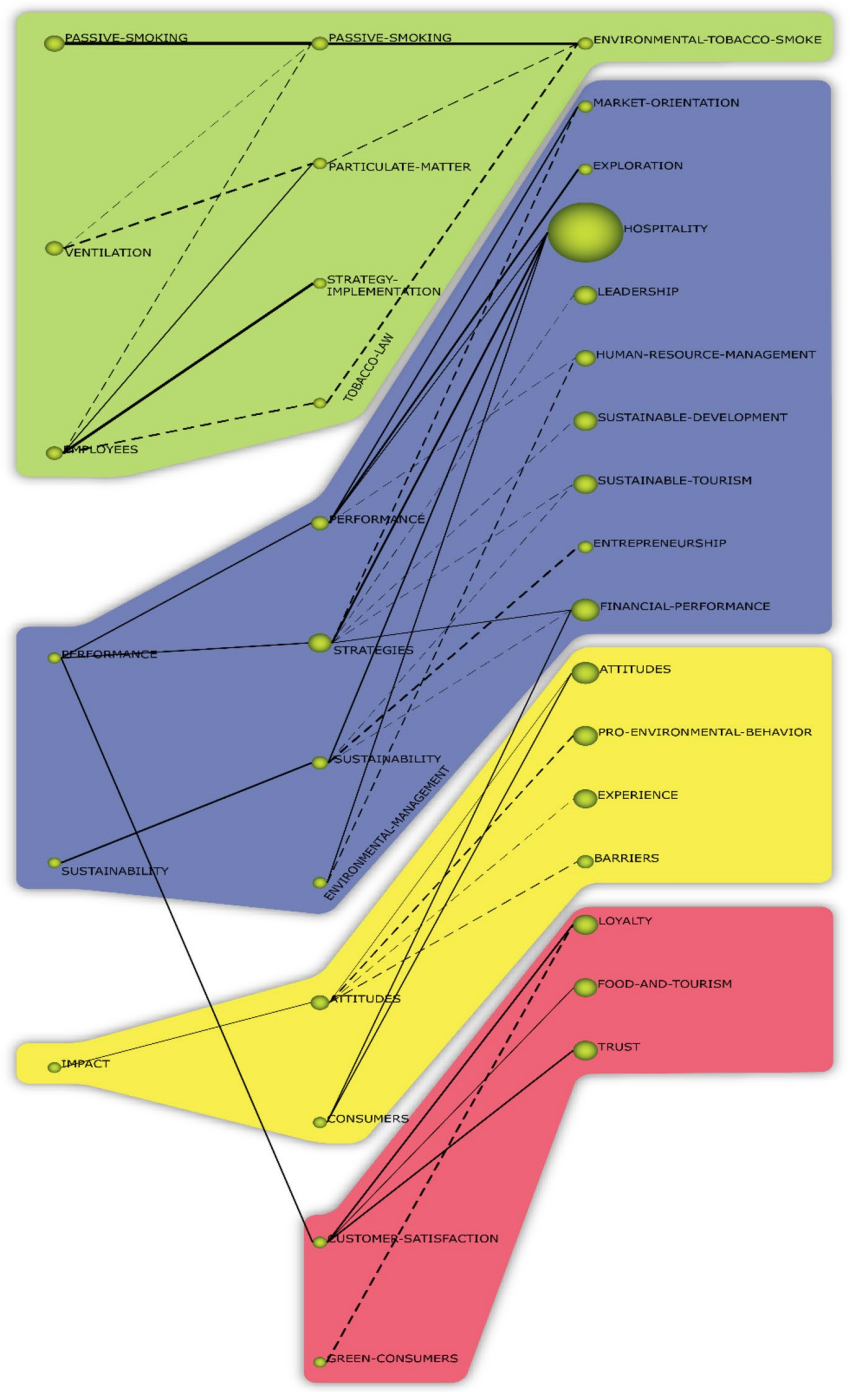
Thematic evolution of environmental knowledge in the hospitality industry. Source: own elaboration

The conceptual evolution map is provided in Fig. 4, where each column is related to a period. There is a link between the themes of two consecutive periods if both themes have keywords in common, indicating a denser connector. The continuous lines in Fig. 4 represent a thematic association. If the line is dashed, it shows that the connected themes share keywords other than their names.

Themes were clustered to explain a particular theme according to the connections between them and the intensity of the connections. Thus, when various themes were predominantly connected by continuous lines, the group of themes in question was considered a thematic area.

## Discussion

The study of the evolution of environmental knowledge in the hospitality industry research themes has shown the main thematic areas in the field. A meticulous evaluation of the thematic areas utilising keywords and linked documents has identified diverse themes included in each period and their evolution. The keywords for each of the thematic areas over the three periods that make up 26 years are shown in Fig. 4. Based on the bibliometric analysis, an increasing number of articles show that the ambit of environmental learning in the hospitality industry has widened and that, in literature, this type of learning is being applied to new aspects of sustainability, knowledge management and the hospitality industry. This also means that environmental knowledge provides knowledge structures to help face new dynamics in organisations.

Consequently, this study can highlight main research gaps and upcoming research trends and this way answer question 4. Therefore, organisational learning must adopt environmental knowledge to help organisations to change and adapt to new situations in the hospitality industry. This continuing trend implies that the number of documents could continue to grow in the future. This idea is also supported by other indicators: a growing interest in environment issues, the rapid deterioration of the environment, changes in legislation (for instance, the 2030 Agenda for Sustainable Development), food waste, the creation of the Green Deal and the health crisis caused by COVID-19 and its impact on the environment (United Nations, 2020).

The keywords for each of the strategic themes during the three periods that together comprise 26 years are given in Figs. 3 and 4, and they represent the thematic evolution of the field. They contribute to highlighting that the field analysed in this bibliometric study is currently of interest and that this interest is growing. Perhaps this subject will cease to be of interest when the environment does not deteriorate, which will be excellent news. Besides, this is an important contribution of this study because the main domain “environmental knowledge” in the literature received different ways to refer to it, such as, green knowledge or sustainable knowledge. This study presents a knowledge orchestration around of the field, it meaning, an orchestration of the words and seminal references of knowledge on environmental knowledge (Rehman et al., 2021; Rohde & Sundaram, 2011).

The area of Health/Passive Smoking/Employees is constituted of key, connected healthcare concepts that apply environmental knowledge from a different perspective to prevent cancer and mitigate or eliminate the negative effects of workplace smoke in public hospitality spaces. In period 2, researchers were interested in studying tobacco laws. Currently, tobacco legislation is very restrictive, and this theme appears as highly developed and isolated. A plausible explanation for this is that the negative effects of tobacco were incentives to changing the laws and making them more restrictive. However, this area is still under study due to researchers continued interest in contributing to developing more sustainable and healthier workplaces (Drope & Chapman, 2001; Mitova et al., 2016). The theme Air Pollution covers research about environmental knowledge applied to ventilation and fine-particulate matter, and the findings of this area could be applied to different contexts, such as the pandemic crisis (Mulcahy et al., 2005). Health in the context of COVID continue been studied from different perspective, such as, from employees perspectives and health in their place works.

The theme Employees/Human resource management mostly includes motor and transversal themes during the three periods. This area includes research related to environmental knowledge applied by workers in different models (implementation of certification, corporate social responsibility, knowledge sharing, skills, capabilities, etc.). The presence of links with sustainability in hospitality should be highlighted. The effect of environmental methods and knowledge agents mainly focus on attitudes. It is also interesting to note that intellectual capital is very important in this area (Martinez-Martinez et al., 2019a, b). In this theme, human capital plays an important role, knowledge transfer (Ferrer-Serrano et al., 2021) and knowledge sharing.

The main focus of Pro-Environmental Behaviour is active behaviour in favour of taking care of the environment from different perspectives: stakeholders, businesses and society (Martínez-Martínez et al., 2021). This is an important finding to encourage researchers and practitioners to continue in this line of work, giving it more visibility and transferring their important findings.

In response to the question of the role of the organisation to maximise the value of environmental knowledge and its management, in line with the study of Rohde and Sundaram (2011), this study proposes that companies in the hospitality industry are “essential and irreplaceable orchestrators of environmental knowledge”. As the evolution of publications shows, companies in the sector have tried to develop different mechanisms and knowledge structures that have made it possible for environmental protection measures not only to be understood by different stakeholders (e.g., customers, employees, or tour operators), but also have helped hospitality companies to generate direct value (e.g., improvement of results and reputation) but also indirect effects such as social value and brand knowledge.

Finally, the Customer Satisfaction area has become essential to improving not only financial performance but also environmental performance (Engström Truls et al., 2003). In hospitality, according to Kang et al. (2012), customers are willing to pay more to have a more sustainable experience. It is important to know what the demands of different customers are in order to satisfy them. Employees are in contact with these customers and can develop relational and human capital. Relational capital can contribute to improving relationships with customers, and human capital, if exploited through relations, can contribute to improving customer loyalty (Cegarra-Navarro et al., 2020; Sardo et al., 2018). Intangibles are very valuable as they can create competitive advantages and are difficult to imitate by competitors (Kaplan & Norton, 2003). Intellectual assets are non-financial indicators of value and measuring, managing and exploiting them should be a key element of any organisation’s strategy (Duffy, 2000; Edvinsson & Malone, 1997). Among intangible assets, client capital can help companies to generate competitive advantages. Client capital is understood as the value of customer relationships and the contribution that this value generates for future company growth perspectives (Duffy, 2000), and it is considered an intangible asset. In today’s world of tight margins, shorter product lifecycles and costly marketing efforts, maintaining profitability is the biggest challenge organisations face. A strong and loyal customer base is critical for business success. From the perspective of knowledge, customer avoid the practices of hiding knowledge, some studies show that stakeholders can contribute, for example, to achieve the objectives of the Government, if Government not hidden knowledge them (Cegarra-Navarro et al., 2021). Consequently, the practices of hidden knowledge should be studied in the context of environmental knowledge in hospitality; it is a gap a possible line of research.

### Contributions to Theory and Implications for Practice

Several theoretical and managerial implications can be drawn from this study. First, from a theoretical perspective, this is the first study on this topic. Besides, through the analyses of the past, development and possible future lines of research in this field, this contribution is central for researchers and practitioners. Indeed, this study provides the themes related with the field and the links among them. Researchers can gather information about theme developments and see which ones have been highly developed or have declined in the last 25 years. Bibliometric analysis can serve as valuable overviews of a topic for planning practitioners who are looking for evidence to guide their decisions and, therefore, their quality can have very real-world implications. In addition, researchers can learn about the motor and emerging themes. In other words, this work contributes to identifying gaps in the academic literature for future lines of research.

The last 25 years selected, and the demanding research criteria allowed us to analyse the contributions with the most significant impact and identify the research agenda. This study enables the scientific community to know the current state of the art, the most and least studied relationships and where the focus should be placed in the future. In this sense, our theoretical arguments make several significant contributions to knowledge management and sustainability literature. Identifying the reference articles on this phenomenon and reflecting on the most salient contributions allows for an extension of the existing state of the art.

Second, this paper offers a holistic and bibliometric view of environmental knowledge in the hospitality industry. This work fills a gap in environmental knowledge research by improving understanding of the determinants, factors and performances of environmental knowledge in the hospitality industry.

Third, this study offers an orchestration of knowledge around the field “environmental knowledge”. It is an interesting contribution to knowledge management field, and also it is interesting for professionals to make decisions and take care of the environment.

Four, one theme present in the three periods is “employees”. This is interesting as it highlights that stimulating a culture of learning and unlearning among employees can improve the current situation of sustainability in the hospitality industry. Managers must motivate employees to share, unlearn or relearn environmental knowledge, structures, concerns and procedures about environmental practices. It is well-known that the hospitality industry is in continuous change and needs to transform some traditional business models to adapt to new requirements from customers and society while preserving the environment. In addition, the increasing deterioration of tourist destinations can be mitigated with the help of environmental learning.

Five, the social and environmental impacts of research in this field have been analysed in this longitudinal study. “Health” and “intellectual capital” (human, structural and relational) were considered in the past and the present, and everything seems to point to the fact that they will be motor themes in the future too. Nowadays, these are two issues that concern people, and as their quality of life is deteriorating, it is necessary to act now (Severo et al., 2021).

Regarding managerial implications, there is broad agreement on the benefits that environmental knowledge creation and use, firstly to pro-environmental behaviour and, secondly, to organisational performance. The role of environmental knowledge is important in value creation and must be dynamically articulated with business strategy of companies. This means that changes in an organisation’s environmental strategy imply adjustments in the environmental knowledge management and environmental strategy. An proactive environmental strategy can suppose the reconfiguration of resources and processes in organisations (Cegarra‐Navarro & Martínez-Martínez, 2017). This process requires an appropriate dynamic combination of social relationships, management practices and technical tools that has a significant effect on business performance; some studies find a positive relation between environmental knowledge and business performances (Fraj et al., 2015; Martínez-Martínez et al., 2015).

Finally, a theoretical and practical implication is the conceptual evolution of environmental knowledge in the hospitality industry, divided into four thematic areas. It is a relevant contribution since researchers can have an overview of the main areas that have been investigated during the last 26 years of environmental knowledge applied to the hospitality industry.

## Conclusion, Limitations and Future Perspectives for Research

In this research, a systematic and highly automated bibliometric method has been followed to analyse the literature on environmental knowledge in the hospitality industry. It is a growing field which is particularly relevant to knowledge management, sustainability and hospitality, where business managers must face the different challenges currently present in the hospitality industry. The recent challenges related to hospitality can be seen to revolve around agents of environmental knowledge. Our longitudinal analysis presents findings with theoretical and practical implications.

Research on environmental knowledge in the hospitality industry is increasing as the number of publications and citations for these papers has grown, especially during the third period (2016–2020). The most productive countries are the United States (USA), followed by Spain, England and China. Significant differences have been found in the topics investigated by the researchers during a period of 26 years. However, some themes are motor and researchers are still investigating them.

The conceptual evolution of the main themes was presented, taking into consideration the results of document content analyses for the three periods, the themes of research and the main concepts which have evolved throughout the years. Keywords that authors used generated some new concepts, while some others fell into disuse. Four thematic areas were presented in this study: Health/Passive Smoking/Employees, Sustainable development, Pro-Environmental Behaviour and Customer Satisfaction. The study is an important contribution to the field because researchers can detect the highly developed or declining themes, motor themes and emerging or declining themes that have appeared during the last 26 years. Value creation is an important aspect for stakeholders; a new trend in research is a collaboration between organisations and customers to develop innovative services and products. The role of environmental knowledge is important in value creation and must be dynamically articulated with business strategy of companies. Besides this work contributing to present a orchestration of environmental knowledge, it is an important contribution for the literature academic and professionals to manage intellectual capital in their companies (Rehman et al., 2021).

Basing the study on literature on environmental knowledge in the hospitality industry, this reveals the following trends and challenges. Different perspectives that involve “employees” have been frequently investigated, pointing out the active role they can play in sustainability and as agents of environmental knowledge in hospitality. “Loyalty” is a way to gain customer loyalty, which means increasing customer awareness and changes in procedures and routines. “Pro-environmental Behaviour” is intensive in relational and human capital, and both are also motor themes. In other words, the hospitality industry has a great impact on the components of intellectual capital: human capital, relational capital and structural capital.

Another motor theme is “sustainable tourism”. Stakeholders now demand more sustainable services, destinations, experiences and food. Corporate social responsibility is an important vector in this link because businesses should be more socially responsible with the environment. “Entrepreneurship” related with environmental knowledge in hospitality appears in Q3 in emerging or declining themes. Eco-entrepreneurship is considered to be a social demand and emerging theme, and therefore, it seems that this might also become an emerging issue.

And “experience” or know-how, which is considered a motor theme, is close to basic and transversal themes. Emerging themes suggest that experience is important. Therefore, the hospitality industry will have to continue learning and unlearning to face new challenges. A possible future line of research in hospitality is the combination of learning with unlearning and relearning.

This study presents the limitations inherent in a theoretical and bibliometric analysis. The analysis should be extended to a quantitative analysis. However, this limitation is an opportunity for new research to focus on understanding the potential impact of environmental knowledge management in the worldwide hospitality industry. This could be an ambitious project, but this work could be the first step towards developing a global project. Another opportunity is to identify how COVID-19 has affected this field and whether it could result in new emerging themes in the future, for example hidden environmental knowledge (Cegarra-Navarro, Vătămănescu, & Martínez-Martínez, 2021) from a double perspective citizen and governments, disabsorptive capacity or relearning.
